# Chagas disease ecoepidemiology and environmental changes in northern Minas Gerais state, Brazil

**DOI:** 10.1590/0074-02760170061

**Published:** 2017-11

**Authors:** Elisa Neves Vianna, Ricardo José de Paula Souza e Guimarães, Christian Rezende Souza, David Gorla, Liléia Diotaiuti

**Affiliations:** 1 Universidade de Brasília, Faculdade de Medicina, Departamento de Patologia, Brasília, DF, Brasil; 2 Instituto Evandro Chagas, Direção Geral, Laboratório de Geoprocessamento, Ananindeua, PA, Brasil; 3 Alô Meio Ambiente e Geoprocessamento Ltda., Belo Horizonte, MG, Brasil; 4 Universidad Nacional de Córdoba, Instituto de Altos Estudios Espaciales Mario Gulich, CONICET, Córdoba, Argentina; 5 Fundação Oswaldo Cruz-Fiocruz, Centro de Pesquisas René Rachou, Laboratório de Triatomíneos e Epidemiologia da Doença de Chagas, Belo Horizonte, MG, Brasil

**Keywords:** Chagas disease, Triatoma, epidemiology, geoprocessing

## Abstract

**BACKGROUND:**

*Triatoma sordida* and *Triatoma pseudomaculata* are frequently captured triatomine species in the Brazilian savannah and caatinga biomes, respectively, and in Brazilian domiciles.

**OBJECTIVES:**

This study identified eco-epidemiological changes in Chagas disease in northern Minas Gerais state, Brazil, and considered the influence of environmental shifts and both natural and anthropogenic effects.

**METHODS:**

Domicile infestation and *Trypanosoma cruzi* infection rates were obtained from triatomines and sylvatic reservoirs during the following two time periods: the 1980s and 2007/2008. Entomological and climatic data with land cover classification derived from satellite imagery were integrated into a geographic information system (GIS), which was applied for atmospheric correction, segmentation, image classification, and mapping and to analyse data obtained in the field. Climatic data were analysed and compared to land cover classifications.

**RESULTS:**

A comparison of current data with data obtained in the 1980's showed that *T. sordida* colonised domiciliary areas in both periods, and that *T. pseudomaculata* did not colonise these areas. There was a tendency toward a reduction in *T. cruzi* infection rates in sylvatic reservoirs, and of triatomines captured in both households and in the sylvatic environment. *T. sordida* populations have reduced in the sylvatic environment, while *T. pseudomaculata* showed an expanding trend in the region compared to counts observed in the 1980's in the sylvatic environment. This may be related to high deforestation rates as well as gradual increases in land surface temperature (LST) and temperatures along the years.

**MAIN CONCLUSIONS:**

Our results suggest a geographical expansion of species into new biomes as a result of anthropogenic and climatic changes that directly interfere with the reproductive and infection processes of vectors.

Chagas disease (CD), also known as American trypanosomiasis, is a parasitic disease caused by *Trypanosoma cruzi*, which is transmitted by the Triatominae (Reduviidae). *Triatoma sordida* is the most frequently captured triatomine in Brazil, including northern Minas Gerais state. This species is also found in the Chaco region of Argentina, Paraguay, and Bolivia (Carcavallo et al. 1999). The vector is endemic in the Cerrado biome, where it invades homes and colonises peridomiciles (Oliveira & Silva 2007). The species has broad distribution in nature, and adult dispersion occurs mainly by the end of the first half of the year (Forattini et al. 1983). In an artificial environment, *T sordida* is ornithophilic and is often found in chicken coops (Diotaiuti et al. 1995a). However, it is an opportunistic species and can feed on humans (González-Brítez et al. 2014). In the sylvatic, it utilises different food sources (Diotaiuti et al. 1993).

*Triatoma pseudomaculata* is another Brazilian autochthonous vector of Chagas disease. It is associated with the Brazilian semiarid region and colonises peridomiciles frequently and extensively (Rossi et al. 2015). Although already recorded in the Brazilian state of Minas Gerais, its occurrence is less relevant than several other triatomine species that occur in the northern part of the state. In the sylvatic, *T sordida* and *T. pseudomaculata* are found under tree bark and crevices, and their life cycles depend on the biology of the sylvatic animals that serve as their food sources (Diotaiuti et al. 1993).

Northern Minas Gerais has boundaries with the Brazilian Northeast, a region with low social-economic levels and low human development index (HDIs-PNUD). Historically, the region has high rates of *T. sordida* infestation (Diotaiuti et al. 1995a). The human prevalence of Chagas disease in the Mato Verde municipality in 1979 was 37.9%, and 100% of analysed dwellings were infested by triatomines (Diotaiuti et al. 1995a). After initiating control programs for *T. infestans* in this region, *T. sordida* persists in colonising both domestic and peridomestic areas (Pires et al. 1999), although the prevalence of the disease has declined to 2.4% nationally since the 2000s (Martins-Melo et al. 2014).

Many studies have reported the distribution of triatomines and their association with land cover and climatic variables (Gurgel-Gonçalves et al. 2012, Medone et al. 2015). Layers of land cover and soil obtained from satellite images can be used to monitor species habitat changes. This approach allows the evaluation of conditions that are favourable for the dispersion of the vector as well as its development, reproductive success, and survival (Omumbo et al. 2002). Climate conditions are evaluated using data such as land surface temperature (LST) and the normalised difference vegetation index (NDVI) (Goetz et al. 2000, Guimarães et al. 2010). In general, the deforestation of large rural areas is associated with changes in climate patterns. These changes may interfere with vector potential and thus have epidemiological importance (Gorla et al. 2002).

The objective of the present study is to identify changes in the ecoepidemiology of Chagas disease in an area of northern Minas Gerais by analysing infestation by triatomines in sylvatic and domestic environments, the presence of reservoirs, infection by *T. cruzi,* and the relationships between these factors and environmental and climatic shifts in the region compared to data obtained in the 1980s by Diotaiuti et al. (1993).

## MATERIALS AND METHODS

### Area of study

**Fig. 1:**
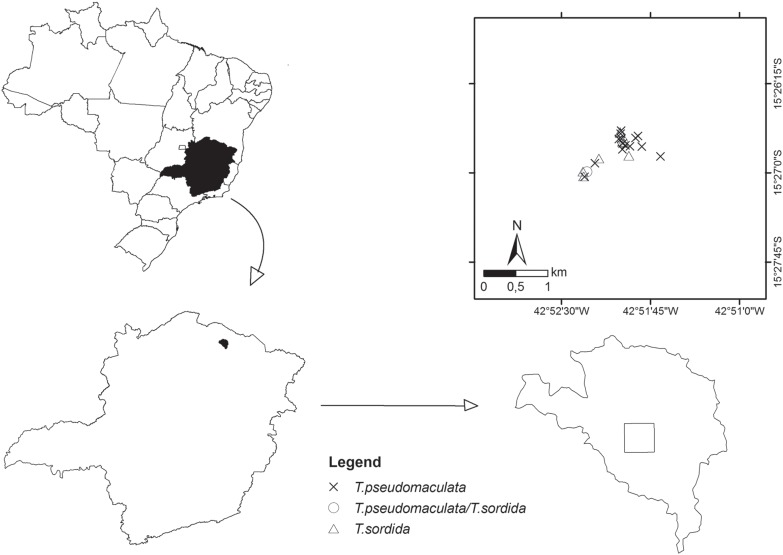
the locality selected for the study in the municipality of Mato Verde, in Jurema, showing the collection points for triatomines in 2007/2008 and maps of Minas Gerais state, Brazil, that were constructed in ArcGIS.

The study was performed in the municipality of Mato Verde, 15° 23′ 49″S, 42° 51′ 57″W, which has an altitude of 550 m and is located in northern Minas Gerais state (Fig. 1). According to the Koppen-Geiger climate classification system, this region is BSw-semiarid (B) and has a mean total annual precipitation between 380 and 760 mm (S) and summer rains (w). The region has two well-defined seasons, including dry (during winter) and rainy (during summer). November, December and January are the wettest months, and June, July and August are the driest. The municipality has an estimated population of 12,921 inhabitants and contained 53 rural localities within 706 km^2^ of territory in 1995. In 1995, the municipality was broken up to create the municipality of Catuti, resulting in a total area of 474 km^2^.

Mato Verde is in an area of ecological transition within the northeastern savanna (Caatinga) that contains dry forest (deciduous forest), savanna (campo cerrado) and Cerrado *sensu strictu.* The deciduous forest presents large trees with up to 50% leaf fall during the dry season.

The rural locality of a municipality is defined by the existence of geographically isolated domiciles surrounding a distant urban area possessing natural or artificial limits. Jurema, a rural locality of the Mato Verde municipality, was selected for this study (Fig. 1). Jurema is located 7 km from the urban area of the municipality (15° 26′ 51.2″S and 42° 51′ 56″W), occupies an area of 20.27 km^2^, and suffered strong effects from cotton plantation activity during the 1980s. At that time, Jurema showed high rates of home infestation by triatomines (Diotaiuti et al. 1993). The locality had 38 and 52 houses in 1985 and 2008, respectively, and included discontinuous forest fragments.

### Data collection: entomological data from the domestic environment

The historical entomological information used to evaluate the occurrence of triatomines and *T. cruzi* infection in the locality of Jurema in the municipality of Mato Verde (according to the methodology recommended by World Health Organization) was gathered from control programme reports. From 1982 to 1986, insect collection in domiciles was carried out semi-annually cycles in all houses of the rural locality, and infested houses were sprayed with BHC (30% hexachlorobenzene). From 1986 to 2000, pyrethroid insecticides were introduced in the control programme, and the cycles of research and spraying became annual. Beginning in the year 2000, 50% of the rural localities were surveyed in one year, and the remaining 50% were surveyed in the following year to make a biennial survey. To sample triatomines, the Chagas Disease Control Program (CDCP) used the man-hour method for 60 min in each domiciliary unit (intra- and peridomicile, using 2% Pirisa^®^ as a dislodge chemical) until 1986.

### Entomological data from the sylvatic environment

During the present study, triatomine collection from the sylvatic environment was carried out in the Jurema locality in July 2007 and in February 2008 in six forest fragments. Collection techniques included active searches and live bait traps. Six transects (one per forest fragment) were marked from the village centre (200 to 400 metres), and searching stations were placed approximately every 10 metres. Branches were cut, and bark was removed using knives and axes, and the captured triatomines were kept in individual pots. Five people participated in the capture, and they worked for 4 h in each forest fragment. In February 2008, surveyed and positive ecotopes were recorded. Fifteen to 20 live bait-traps (Noireau et al. 2002) were also distributed among the ecotopes (two to three per ecotope) using a mouse as bait. For purposes of comparison, the data on triatomine captures were used for forest fragments in the same rural location from the surveys completed between September 1985 and 1986. These data were obtained through a study by Diotaiuti et al. (1993), which included an active search of stones and a dissection of live or dead tree trunks and tree bark. On that occasion, the captures were carried out by two people.

During the 2007-2008 period of the present study, potential *T. cruzi* reservoirs (sylvatic mammals) were captured in the same forest fragments that were surveyed between 1985 and 1986 by Diotaiuti et al. (1993). Tomahawk^®^ and cage-like traps were placed that used grains, banana, dried meat, and cod-liver oil as bait. Twelve traps were distributed over six forest fragments, and they were linearly arranged approximately 10 m apart. Each was placed for two weeks at the end of the afternoon and then surveyed the following morning for five consecutive days. The captured sylvatic animals were anaesthetised using 2.5% sodium thiopental and submitted to xenodiagnoses. Ten 3rd or 4th-stage *Rhodnius neglectus* nymphs were used for each animal (Forattini et al. 1976). A sample of each species of captured animal was culled (authorised by the Brazilian Institute of Environment and Renewable Natural Resources IBAMA - license number 11019-1, registration number 1908597). Animal skulls and skins were identified at the Museum of Natural Sciences of the Catholic University of Minas Gerais. The number of sylvatic mammals and the rate of natural infection for *T. cruzi* were compared with the data collected by Diotaiuti et al. (1993).

### Triatomine identification and T. cruzi infection

Adult triatomines were identified according to Lent & Wygodzinsky (1979), and the nymphs were morphologically compared with standard specimens maintained in the collection of the Centro de Pesquisas René Rachou/FIOCRUZ (René Rachou Research Centre/Oswaldo Cruz Fundation).

Each live insect was submitted to abdominal compression to verify the presence of protozoan flagellates. The parasitological test was performed in fresh triatomine faeces under an optical microscope at 40X and 100X. Triatomine infection with *T. cruzi* was confirmed using multiplex polymerase chain reaction (PCR) (Liarte et al. 2009).

Diotaiuti et al. (1993, 1995b) described capture rates and triatomine infection in the sylvatic as well as the identification of reservoirs that were found to be positive for *T. cruzi* in this locality in the 1980s using zymodeme analysis. Past and present data were subjected to qualitative and quantitative comparison using Fisher's exact test.

### Environmental and spatial analysis

Environmental modifications in the region were evaluated using data related to land use and land cover and soil surface temperature using Landsat image processing. They were estimated specifically for the chosen rural locality. For the entire municipality, data were gathered from crop-livestock and demographic censuses for the years 1980, 1985, 1996 and 2006. The data included the area planted with cotton cultivation, the quantity of each farm animal, the area used as pasture, and the area covered by natural forests. Data were obtained from the Instituto Brasileiro de Geografia e Estatística (IBGE) (Brazilian Institute of Geography and Statistics).

A global positioning system (GPS) receiver (Garmin eTrex^®^) was used to obtain the spatial coordinates of *Triatoma* spp. breeding sites in July 2007 and February 2008. The georeferenced database was processed using SPRING software (http://www.dpi.inpe.br/spring/), and the appropriate ArcGIs (http://www.esri.com/) algorithms were applied on the Landsat imagery to carry out the classification and atmospheric correction, segmentation, and mapping and to analyse the data obtained in the field. The results are shown in the form of thematic maps.

Images from the satellite LANDSAT TM 5 that were obtained on 08/07/1984, 04/06/1989, 18/06/1994, 31/05/1989, 16/06/2005 and 26/07/2008 were collected from the catalogue of images from the National Institute for Space Research with the objective of creating maps of land use and land coverage. Colour images were obtained using composition RGB 543. The atmospheric correction was performed by applying contrast.

The applied method for the classification of the images was segmentation in SPRING software version 4.3.3. The pixel groups were analysed with a similarity of 10 and five for minimal pixel area. After the homogeneous regions were created, a non-supervised method (Isoseg) was applied that allowed the software to identify the maximum possible classes from the specified parameters. The result of the classification was converted from raster to vector and run in the program ArcGIS 9.3 to define the final classes according to the floristic survey. The eight following classes of land use and coverage were considered: rocky outcrop, dry forest in regeneration, crop area, Cerrado, riparian forest, dry forest, exposed soil, and pasture (Fig. 3). SPRING software was also used to compare the classified images from 1984 and 2008 using the LEGAL language. LEGAL is a Map Algebra tool that compares pixel by pixel differences in vegetation between two time points. Therefore, any modification that occurs in the vegetation will produce a response as either in the same class (i.e., no modification occurred) or in a different class (i.e., modification occurred).

**Fig. 2:**
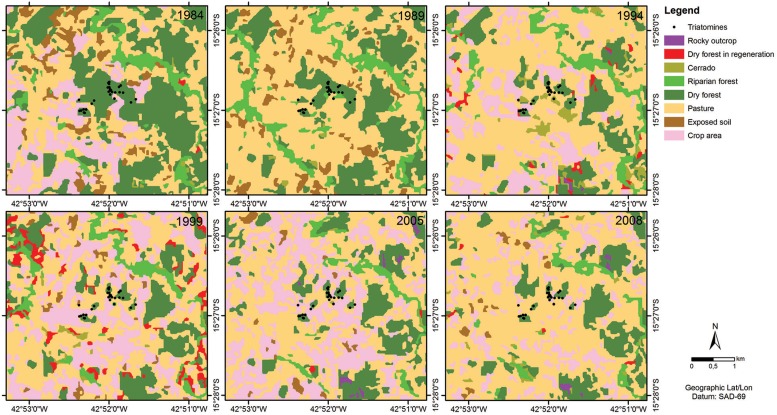
images classified from Landsat satellite imagery, including retrospectives of Jurema, in Mato Verde, Minas Gerais state, Brazil, that represent land use and land cover and breeding sites of insects collected in 2007/2008. (A) 1984; (B) 1989; (C) 1994; (D) 1999; (E) 2005; (F) 2008. For the matches between colours and classes, see Fig. 3.

**Fig. 3:**
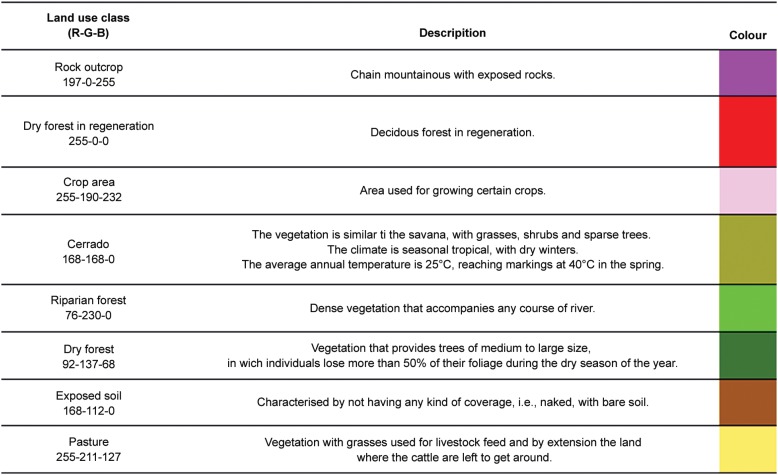
definitions of the land use and land cover classes used in the study.

A total of eight classes were used to evaluate changes in land use and land cover in the study period (Fig. 2). The eight classes were grouped into three classes for a better comparison (Fig. 3). “Same class” was used to represent the classes that did not change between years 1984 and 2008. Changes were divided into “another class” and “deforestation” classes. The “deforestation” class indicated changes from one vegetation class (i.e., Cerrado, riparian forest or dry forest) to another nonvegetation class (i.e., rocky outcrop, crop area, exposed soil or pasture). To diagnose vegetative modifications, a Pearson's correlation test was applied to compare the area in square metres that was occupied by each class.

To evaluate changes in the temperature of the rural locality during the study period, the thermal band of Landsat images was used to estimate the surface temperature. For these analyses, we extracted pixel values for minimum, average, and maximum land surface temperature (°C) during the study period.

For the transformation of pixel intensity (0-255), we used the quadratic regression model described by Malaret et al. (1985) (Equation 1), which is included in ArcGIS 9.3. The equation is shown below:

T = 209.831 + 0.834DN - 0.00133DN2, where: T = apparent temperature in Kelvin (K), and DN = the digital number of each pixel.

As a final step, Kelvin degrees were converted to Celsius (°C).

To assess possible changes in the local climate, normal climatic measurements from the years 1979-2008 were made by the Brazilian Instituto Nacional de Metereologia (INMET) (National Institute of Meteorology) at the Brazilian meteorological station in Monte Azul, which is located 25 km from the municipality. Data were considered for the periods 1979-1982 and 1990-2008, and the series data were obtained for the drier and wetter months for the following variables: precipitation (mm), minimum temperature (°C), maximum temperature (°C), and humidity (%). The means for climate variables were calculated for months with values that were available for each year except for precipitation, which was calculated as the sum of the values of the months. The years that had three or fewer values (for months) were excluded from the analysis. A simple linear regression was performed using the climate data means as the response variable (y) and the year as the explanatory variable (x). All analyses were performed using SPSS 15.0.

## RESULTS

Table I describes the temporal changes in infestation observed in rural homes in Jurema between 1982 and 2014. Changes in the triatomine population were observed over the years after *T. infestans* infestations were eliminated, and the last specimen was captured in 1987. *T. sordida* was the dominant species, and it persists throughout the years. *T. pseudomaculata* was extremely rare (one specimen was identified in 1990). The rate of *T. cruzi* infection in triatomines fluctuated over time for the species.

**TABLE I:** Infestation, number of insects, infection by *Trypanosoma cruzi* of triatomines captured in the domiciliary units (DUs) of the rural locality of Jurema, city of Mato Verde, Minas Gerais state, Brazil, in the 1982-2014 period

Year	DUs surveyed	N° of infested DUs/II (%)	*Triatoma infestans*	*T. sordida*	NIR (*T. infestans*)	NIR (*T. sordida*)
1982	34	13 (38.2)	14	51	0	0
1983	37	17 (45.9)	14	81	15.4	0
1984	39	7 (17.9)	4	43	33.3	0
1985	38	14 (36.8)	20	66	6.25	4.3
1986	41	13 (31.7)	10	57	25	3.5
1987	44	13 29.4	6	91	0	0
1988	42	5 (11.9)	0	13	–	0
1989	44	19 (43.2)	0	69	–	0
1990	50	18 (36)	0	58	–	0
1991	51	19 (37.3)	0	58	–	11.3
1992	51	25 (49)	0	62	–	0
1993	49	10 (20.4)	0	25	–	0
1996	50	31 (62)	0	105	–	0
2005	46	54.3(25)	0	52	–	0
2007	44	21 (47.7)	0	142	–	1.8
2009	56	55.4(31)	0	60	–	0
2014	56	42.9 (24)	0	53	–	0

II: infestation index (number of positive domiciliary units for triatomines/number of domiciliary units inspected x 100). NIR: natural infection rate = number of positive triatomines for *T. cruzi,* divided by the number of examined triatomine X 100. Source: FUNASA-Ministry of Healthy. One exemplar of *Triatoma pseudomaculata* in 1990 is not included in the Table.

In the sylvatic, there were fewer ecotopes infested by triatomines in 2007/2008 (Table II) than in 1985/1986 (χ^2^, p ≤ 0.0001). The total number of *T. pseudomaculata* and *T. sordida* captured in both periods differed (Fisher's exact test, p < 0.0001), and there was a reduction in the number of captured *T. sordida* and an increase in the number of captured *T. pseudomaculata.* The average number of insects captured per ecotope was 1.83 for 1985/86 and 0.73 for 2008, and the difference between the two is not statistically significant. There was a trend towards a reduction in the *T. cruzi* infection rate in the triatomines and reservoirs, although the result of Fisher's exact test was not significant for either triatomine infection (p = 0.116) or the reservoirs (p = 0.159) in either survey period (Table III). In the 1985/1986 period, all infected mammals belonged to the species *Didelphis albiventris.* In 2007, out of 11 captured mammals, one *D. albiventris* was infected (captured in the forest, 500 m from a house) as was one *Cerradomys sp.* (captured in a house barn) (see Table III). No triatomines were caught by live bait traps.

**TABLE II:** Rural locality of Jurema, Mato Verde, Minas Gerais state, Brazil. Triatomines caught in the wild during 1985/1986 and 2007/2008

Research period	Days worked	Ecotopes researched	Ecotopes infested	*Triatoma sordida*	*T. pseudomaculata*	Total
September-August 1985/1986	35	105	37 (35.2%)	68 (97.1%)	2 (2.9%)	70 (100%)
July/2007	5	*	10	5 (29.4%)	12 (70.5%)	17 (100%)
February/2008	5	332	19 (5.7%)	14 (53.8%)	12 (46.2%)	26 (100%)

* ecotopes researched but not counted.

**TABLE III:** Parasitological examination of mammals and triatomines infected with *Trypanosoma cruzi* in the wild in the Brazilian locality of Jurema, Mato Verde, Minas Gerais state

	N° of mammals	N° of mammals infected	N° of collected triatomines/examined	N° of triatomines infected
1985-1986	37*	15 (40.5%)	68/64	7 (10.9%)
2007-2008	11**	2 (18.2)	26/22	0

* 30 *Didelphis albiventris* and seven *Thrichomys apereoides*;

** three *D. albiventris,* seven *T. apereoides* and one *Cerradomys sp.*

Table IV shows the data from the crop-livestock census of the municipality for 1980, 1985, 1996, and 2006. There was an increase in pasture area, a reduction in forest area (e.g., Cerrado, deciduous seasonal forest, riparian forest, and deciduous seasonal forest in regeneration), a reduction in the area encompassing cotton crops, a reduction in cattle in the region, and a slight increase in goat numbers, whereas poultry numbers remained stable. Fig. 2 shows the results of the analysis of land use and land coverage estimated using satellite images in the years with the respective classes for the rural locality, and Fig. 3 shows the definition of the vegetation classes used in the study. The LEGAL analysis revealed deforestation and changes in deciduous forest, riparian forest and Cerrado in open areas (Pasture and crop areas). Table V shows the changes in the percentages of the classes between 1984 and 2008 and highlights the most relevant ones (six classes). A total of 19.9% of the area occupied by deciduous seasonal forest, Cerrado and riparian forest was converted into pasture between 1984 and 2008, 0.03% was converted into exposed soil, and 2.8% was converted into crop areas, for a total of 22.8% deforestation in the area.

**TABLE IV:** Data from the Instituto Brasileiro de Geografia e Estatística (Brazilian Institute of Geography and Statistics) Agricultural Census of use and land cover in the municipality of Mato Verde, Minas Gerais state, for period between 1980 and 2006

Year	Pastures (ha)	Woods (ha)	Cattle (n)	Goats (n)	Poultry (n)	Cotton farming (ha)
1980	10.229	23.556	25.000	300	32.000	18.970
1985	8.988	17.149	22.666	250	26.356	18.000
1996	8.358	11.650	28.950	250	29.700	4.650
2006	24.459	9.919	13.605	2.050	27.956	1.300

ha: hectares; n: number of animals.

**TABLE V:** Matrix showing the difference between the 1984 and 2008 classifications (%) using LEGAL language

1984\2008	Cerrado	Riparian forest	Dry forest	Pasture	Exposed soil	Crop area	Total
Cerrado	0.021	0.056	0.235	0.077	0.000	0.005	0.394
Riparian forest	0.407	2.611	0.51	2.929	0.003	0.139	6.599
Dry forest	0.612	0.288	13.453	16.932	0.022	2.657	33.96
Pasture	0.380	0.758	2,003	24.521	0.651	6.991	35.3
Exposed soil	0.009	0.025	0.038	4.659	0.197	1.134	6.06
Crop area	0.128	0.137	0.281	12.989	0.379	3.771	17.69
Total	1.557	3.875	16.52	62.107	1.252	14.697	100

Spearman's rank correlation coefficient identified a statistically significant and negative correlation between the changes that occurred throughout the years between the forest (deciduous and riparian forest) and the crop area (pasture and open areas) (r = -0.885, p = 0.019).

Table VI shows the LST values for 1984, 1989, 1994, 1999, 2005 and 2008. The LST mean ranged from 21.42°C in 1984 to 28.5°C in 2008, with the highest value of 32.39°C recorded in 1999. Analyses using Pearson's and Spearman's correlation tests between the vegetation and LST classes did not find statistically significant results.

**TABLE VI:** Land surface temperature (LST) in degrees celsius (°C) for Jurema, Minas Gerais state, in the years 1984 to 2008

	Minimum	Mean	Maximum	SD
1984	16.46	21.42	25.09	1.6
1989	23.13	28.33	32.08	1.63
1994	22.13	27.68	32.08	1.63
1999	27.01	32.39	37.67	1.76
2005	20.1	24.75	27.96	1.35
2008	21.2	28.5	33.41	1.92

SD: standard deviation.

Table VII shows the maximum and minimum temperatures in the dry and wet seasons, demonstrating a trend toward an increase between seasons.

**TABLE VII:** Results of the linear regression test between the values of maximum and minimum temperatures aggregated by months of the dry season (D) and rainy season (R). Monte Azul Meteorological Station, Minas Gerais state, Brazil

	Regression coefficient (β)	Standard error	T-value	Pr(>|t|)
Year (Tmin R)	0.033	0.008	4.163	5.68e-05
Year (Tmin D)	0.044	0.0145	2.952	0.008
Year (Tmax R)	0.051	0.017	3.029	0.003
Year (Tmax D)	0.044	0.014	3.128	0.002

For each year considered in the region, there was an increase of 0.033°C in the monthly minimum temperature in the dry season months. For the wet season, there was an increase of 0.044°C. The maximum temperature in the wet season increased by 0.051°C, and in the dry season, it increased by 0.044°C according to a regression coefficient, as shown in Table VII. All coefficients were statistically significant. There were no significant differences in analyses involving precipitation and humidity variables (data not shown).

## DISCUSSION

The present study reveals new information regarding the ecoepidemiology of CD in a municipality in northern Minas Gerais. We compared two different time periods (1985/86 and 2007/08) and found that *T. sordida* continues to colonise domiciles, and in the sylvatic environment its abundance was reduced, as were the number of ecotopes infested. The abundance of *T. pseudomaculata* has increased, while environmental changes occurred simultaneously in the region and were mainly associated with vegetation and temperature. There was a reduction in riparian forest, Cerrado, and deciduous forest and an increase in pasture, crop, and exposed soil areas.

The CDPC started in 1982 after *T. infestans* elimination from the municipality. *T. infestans* was eliminated from domiciliary units in 1986, although this did not occur with the native species, *T. sordida*. The existence of complex peridomiciles in the locality may be a fundamental factor in the maintenance of colonisation by *T. sordida*, which finds shelter among different structures, crevices, and holes, hindering its control (Diotaiuti et al. 1995a). Peridomiciliary infestation by this species in the Brazilian states of São Paulo, Minas Gerais, and Goiás was associated with the presence of animal shelters built with wood (e.g., chicken coops and corrals), piles of firewood or wood, and food storage (Diotaiuti et al. 1993, Rossi et al. 2015), as was also observed in the present study region.

In the domicile units of Jurema, the natural *T. cruzi* infection rate fluctuated between 1.8 and 11.3%, probably due to the presence of triatomines and sylvatic reservoirs, which introduce the parasite into the transmission cycle near dwellings. Infected *Cerradomys sp.* and *D. albiventris* were found in peridomicile areas and sylvatic environments, respectively, demonstrating the synanthropic behaviour that is frequently observed in agricultural areas (Pereira & Geise 2007). This provides a link between sylvatic and artificial environments.

A comparison of the present data with those of Diotaiuti et al. (1993) demonstrates that important changes have occurred in the sylvatic environments of the Jurema locality, including decrease in the number of infected triatomines and reservoirs, the number of positive ecotopes, and the prevalence of triatomine species. Data for 2007/2008 captures demonstrated a predominance of *T. pseudomaculata* over *T. sordida*, while the data obtained in 1985/1986 showed that the most abundant species was *T. sordida. T. pseudomaculata* presents a wide distribution in the Brazilian semiarid region (endemic area), predominantly in the Caatinga, and reaches the transition areas of savanna/Caatinga in northern Minas Gerais. In the northeast part of Brazil, its behaviour is like that of *T. sordida* in that it colonises mainly peridomiciles but can also colonise intradomiciles (Assis et al. 2009). This similarity is maintained in the sylvatic environment, where it is found in hollows and under tree bark (Carbajal de la Fuente et al. 2007), which probably furthers the expansion of *T. pseudomaculata* to new territories where conditions are more favourable. This process was observed in Berilo, Minas Gerais, where *T. pseudomaculata* began to predominate over *Panstrongylus megistus*, a species traditionally associated with regions with higher humidity, probably due to increased soil aridity, which favours caatinga species (Assis et al. 2007).

The sites sampled in fragments of the Jurema forest are classified as a mosaic of deciduous seasonal forest because their structural and floristic composition includes elements of Caatinga vegetation and Cerrado. The present study shows that deforestation has increased over the course of the study period. Deforestation produced open areas (Pasture and crop areas). The reduction of *T. sordida* in the sylvatic environment and the reduction in the percentage of ecotopes infested with triatomines observed since the 1980s may be related to the decrease in habitat available for hosts, which are caused by reductions in the area covered by deciduous forest, Cerrado and riparian forests.

There was a change in mean LST in the region between the years 1984 and 2007-2008, probably because of an increase in crop area, exposed soil, and pasture. Some studies of climatic alterations in South America have shown that during the last 50 years, soil surface temperatures have increased by 0.75°C, whereas minimum air temperatures have increased up to 1°C (IPCC 2011). One factor that some authors suggest contributed to these climate alterations in certain regions is deforestation, as shown in the present work. When there is an increase in the micro-climate temperature, there are increases in the triatomine egg eclosion rate, vector reproduction, and dispersion (Curto de Casas et al. 1999), as has already been shown in mathematical models of the occurrence of *T. infestans* (Gorla 2002). These environmental changes may partially explain the sylvatic expansion of *T. pseudomaculata,* a species typical of semiarid climate and vegetation.

The combined use of GIS and statistical techniques indicates the occurrence of significant changes in land cover, especially those related to increases in local pasture at the expense of native forest, as well as significant changes in LST. The LEGAL analysis allows researchers detect transformations in land use and land coverage classes and is more accurate than values obtained directly from satellite images (Barbosa 1997). The algorithm revealed the extent of deforestation that had occurred in the locality.

The density of *T. sordida* in sylvatic ecotopes is characteristically low. A comparison between the data described in Diotaiuti et al. (1993) and the present study indicates that the number of specimens per positive ecotope has varied between one and three insects. Forattini et al. (1983) periodically surveyed over 700 ecotopes in an area of Cerrado and captured only 76 specimens of *T. sordida* in a year. Noireau et al. (1999) captured *T. infestans* and *T. sordida* in Bolivia using live bait traps in tree hollows and obtained a density of individuals of 1.3 to 1.8 per ecotope sampled. The sylvatic ecotopes used by *T. sordida* are considered “unstable” in that they are subject to greater microclimate variation and seasonality of food sources (Diotaiuti 2009). Therefore, the observed climate changes are expected to alter ecotope microclimate and to favour the occurrence of *T. pseudomaculata,* specifically where there is lower humidity and higher temperatures. The results presented here may also be useful for assessments of environmental integrity because the effects of changes in triatomine ecology probably occur in parallel with effects on other organisms and involve unknown consequences for the natural environment.
